# Nitrate and Inhibition of Ruminal Methanogenesis: Microbial Ecology, Obstacles, and Opportunities for Lowering Methane Emissions from Ruminant Livestock

**DOI:** 10.3389/fmicb.2016.00132

**Published:** 2016-02-12

**Authors:** Chengjian Yang, John A. Rooke, Irene Cabeza, Robert J. Wallace

**Affiliations:** ^1^Buffalo Research Institute, Chinese Academy of Agricultural SciencesNanning, China; ^2^Scotland’s Rural CollegeEdinburgh, UK; ^3^Rowett Institute of Nutrition and Health, University of AberdeenBucksburn, UK

**Keywords:** animal health, animal performance, greenhouse gas, nitrate reduction, nitrite

## Abstract

Ruminal methane production is among the main targets for greenhouse gas (GHG) mitigation for the animal agriculture industry. Many compounds have been evaluated for their efficacy to suppress enteric methane production by ruminal microorganisms. Of these, nitrate as an alternative hydrogen sink has been among the most promising, but it suffers from variability in efficacy for reasons that are not understood. The accumulation of nitrite, which is poisonous when absorbed into the animal’s circulation, is also variable and poorly understood. This review identifies large gaps in our knowledge of rumen microbial ecology that handicap the further development and safety of nitrate as a dietary additive. Three main bacterial species have been associated historically with ruminal nitrate reduction, namely *Wolinella succinogenes*, *Veillonella parvula*, and *Selenomonas ruminantium*, but others almost certainly exist in the largely uncultivated ruminal microbiota. Indications are strong that ciliate protozoa can reduce nitrate, but the significance of their role relative to bacteria is not known. The metabolic fate of the reduced nitrate has not been studied in detail. It is important to be sure that nitrate metabolism and efforts to enhance rates of nitrite reduction do not lead to the evolution of the much more potent GHG, nitrous oxide. The relative importance of direct inhibition of archaeal methanogenic enzymes by nitrite or the efficiency of capture of hydrogen by nitrate reduction in lowering methane production is also not known, nor are nitrite effects on other members of the microbiota. How effective would combining mitigation methods be, based on our understanding of the effects of nitrate and nitrite on the microbiome? Answering these fundamental microbiological questions is essential in assessing the potential of dietary nitrate to limit methane emissions from ruminant livestock.

## Introduction

Until relatively recently, the main driver for research on ruminal nitrate metabolism was nitrate poisoning and nitrite toxicity. Nitrate accumulates in certain plants, particularly weeds, that grazing ruminants might consume (Dawson et al., 1997). In some forages nitrate comprises up to 37% of total nitrogen (Miyazaki, 1977). The metabolism of nitrate in the rumen leads to the formation of nitrite which, when absorbed across the rumen wall, reacts with hemoglobin in the erythrocyte to form methemoglobin, which does not carry oxygen. Thus, ruminants grazing nitrate-containing feedstuffs risk illness or even death from methemoglobinemia (Ishigami and Inoue, 1976). In some early studies of ruminal nitrate metabolism, it was noted in passing that nitrate was a potent inhibitor of methanogenesis by the mixed ruminal microbiota *in vitro* (Jones, 1972; Allison and Reddy, 1983). Mitigation of methane emissions is now a high research priority in ruminant research (Martin et al., 2010; Hristov et al., 2013a,b), because ruminants produce large volumes of methane, which is 28 times as potent as carbon dioxide as a greenhouse gas (GHG) (Pachauri et al., 2014). The ability of nitrate to inhibit methane production and its potential as a feed additive is thus being revisited for environmental reasons. This review identifies gaps in knowledge with regard to ruminal microbial ecology, methanogenesis and nitrate metabolism that, if filled, would enable a more comprehensive assessment of the merits of nitrate as a feed additive to decrease methane production.

## Mitigation of Methane Emissions and Possible Role for Nitrate as a Dietary Additive

Ruminal methane emissions represent both a loss of 2–12% of the feed gross energy to the animal (Johnson and Johnson, 1995) and a source of GHG that leads to 37% of total GHG from agriculture in the UK (Department of Energy and Climate Change, 2015). Globally, methane emitted from livestock contributes up to 40% of global anthropogenic methane emissions (Key and Tallard, 2012). Several chemicals that inhibit methanogenesis directly have been evaluated for their efficacy to inhibit enteric methane production in ruminants. These include halogenated hydrocarbons, lipids, and plant compounds such as tannins and saponins (Hook et al., 2010; Morgavi et al., 2010; Hristov et al., 2013a,b). An amusing demonstration of chemical inhibition of methanogenesis occurred when a throat lozenge was added to the ovine rumen, lowering methane production by about two–thirds (Basmaeil and Clapperton, 1978). The reason for this surprising effect was that the lozenge contained chloroform to provide pain relief (today’s product no longer contains chloroform, we have been assured), and chloroform is a structural analog of methane. The downside of many inhibitors of this type is that they often exert adverse effects on feed intake, digestion, and rumen fermentation when added at concentrations high enough to achieve substantial decreases in methane production, while they result in little inhibition of methane production when added at concentrations that do not reduce animal productivity or feed digestion (Hook et al., 2010; Martin et al., 2010; Morgavi et al., 2010; Patra and Yu, 2012). 2, 2, 2-Trichloroacetamide (Trei et al., 1971), hemiacetal of chloral and starch (Trei et al., 1972), bromochloromethane (McCrabb et al., 1997), and anthraquinone (Kung et al., 2003) are effective inhibitors of methanogenesis, but they have other problems, such as cost, legislation, toxicity, volatility, or accumulation in meat which preclude their practical use. The effectiveness of some inhibitors also tends to be transient in nature, because the rumen microbiota adapts around them (Hook et al., 2010; Martin et al., 2010). Although more promising inhibitors are now being developed by rational design (Leahy et al., 2010; Attwood et al., 2011; Romero-Perez et al., 2014, 2015), the problem of the almost infinite ability of microorganisms, particularly complex communities like the rumen, to adapt may limit the usefulness of specific chemical inhibitors. A recently discovered molecule, 3-nitrooxypropanol, may prove to be the exception. 3-Nitrooxypropanol inhibited methane emissions from dairy cows by 30% with no apparent adaptation against efficacy over a 12-weeks period (Hristov et al., 2015). 3-Nitrooxypropanol, which is a structural analog of coenzyme M, inhibits methyl coenzyme-M reductase, the enzyme that catalyzes the last step of methanogenesis. Equally importantly, 3-nitrooxypropanol had no detrimental effects on milk production or feed intake, and indeed increased milk protein and lactose concentrations. Time will tell if these early observations prove to be the major advance that is hoped for. However, even if 3-nitrooxypropanol does fulfill its promise, there remains 70% of methane emissions to be tackled.

In a study of different hydrogen donors that might enhance nitrate metabolism, Jones (1972) observed that nitrate lowered methane production in bovine ruminal digesta *in vitro*. Allison and Reddy (1983) noted a similar effect in a continuous culture system inoculated with ovine ruminal digesta. The results of Iwamoto et al. (1999, 2001a) with goat ruminal digesta indicated the same, though the authors found toxicity to the microbes as measured by lower volatile fatty acid (VFA) production. These findings were instrumental in stimulating *in vivo* experiments to evaluate the usefulness of nitrate as a methanogenesis-inhibiting feed additive/ingredient.

Nitrate has been evaluated in a number of methanogenesis studies both *in vitro* (Sar et al., 2005b,c; Leng, 2008; Guo et al., 2009; Lin et al., 2011, 2013b; Shi et al., 2012; Patra and Yu, 2013) and *in vivo* (Nolan et al., 2010; Morgavi et al., 2010; van Zijderveld et al., 2010, 2011a; Hulshof et al., 2012; Li et al., 2013; Lee and Beauchemin, 2014; Newbold et al., 2014). The results have been among the most promising of all the interventions investigated to date (Hristov et al., 2013a), yet variations in response, e.g., in relation to the basal diet (Troy et al., 2015), are difficult to explain (see the excellent review by Lee and Beauchemin, 2014), particularly in microbiological terms.

Another property in favor of nitrate as a feed additive is that it can have nutritional benefits associated with protein nutrition additional to those deriving from lower methane emissions. Nitrate-N can ultimately be converted to ammonia-N, which is the main N substrate for rumen microbial protein synthesis (Leng and Nolan, 1984), thus the additive can be incorporated as a non-protein-N (NPN) source for the growth of ruminal bacteria, in much the same way as urea (Erfle et al., 1978). Indeed, it has been reported that nitrate is a superior form of NPN compared to urea *in vitro* (Guo et al., 2009). Nitrate reduction is thermodynamically favorable (Guo et al., 2009) and will be linked to ATP synthesis by electron transport-linked phosphorylation in some microbial species (Berks et al., 1995; Iwamoto et al., 2001b, 2002; Yoshii et al., 2003), which would increase the growth yield of nitrate reducing organisms and the overall flow of microbial protein from rumen fermentation. Thus, using nitrate to lower methane emissions may also enable economically favorable reformulation of the N content of the diet, enabling the proportion of expensive protein supplements to be decreased. It should be noted that this does not apply to situations for example where temperate forages are grazed and in which N supply to the rumen is in excess; in this situation, use of nitrate would lower the efficiency of N utilization.

## Nitrate Metabolism in the Rumen and Nitrite Toxicity

Dietary nitrate has been of interest to ruminant nutritionists for many decades (Holtenius, 1957; Allison and Reddy, 1983). Nitrate tastes bitter, which lowers palatability of nitrate-based diets and may cause lower feed intake or feed sorting (Miyazaki, 1977; Lee et al., 2015b), but it is the high nitrate composition of certain plants, such as sugar beet leaves and certain grasses, and the subsequent poisoning of animals consuming these plants that has been of greatest concern (Miyazaki, 1977; Dawson et al., 1997). The reduction of nitrate and accumulation of nitrite in the rumen were first detected by Sapiro et al. (1949) and Lewis (1951), with the observations being followed up in detail by Holtenius (1957), Jamieson (1959), and Wang et al. (1961). Nitrite is absorbed across the rumen wall into the blood where it interacts with hemoglobin in the erythrocyte to form methemoglobin (Lewis, 1951; Holtenius, 1957; Jamieson, 1959), which is incapable of carrying oxygen. The nitrite arising from nitrate reduction is therefore toxic and the consequences of nitrate can be fatal (Cockburn et al., 2013). A variety of other pathological changes may also result from chronic exposure to nitrite (Bruning-Fann and Kaneene, 1993).

**FIGURE 1:**
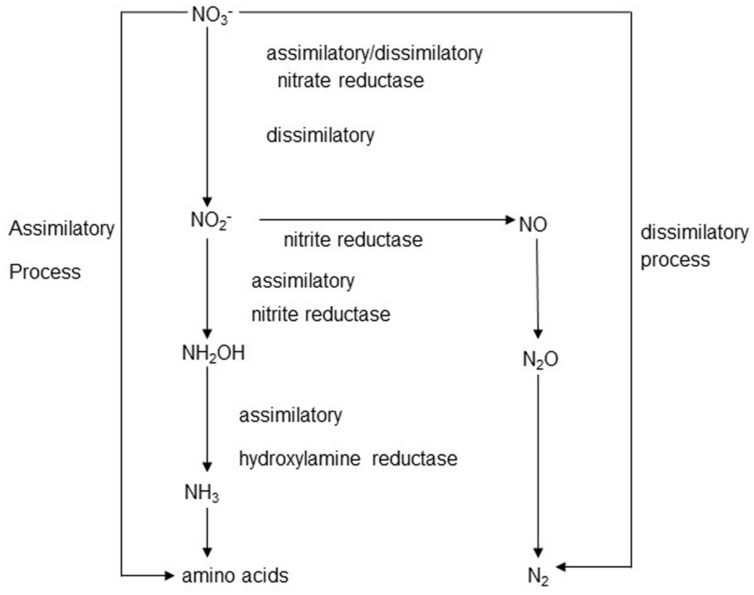
**The assimilatory and dissimilatory routes of nitrate/nitrite metabolism**.

The overall scheme of nitrate metabolism in the rumen is shown in **Figure 1**. Both assimilatory nitrite reduction, leading to ammonia production, and dissimilatory nitrite reduction were shown to occur in rumen contents (Jones, 1972; Kaspar and Tiedje, 1981). In incubations with bovine ruminal digesta *in vitro*, assimilatory nitrate reduction was predominant, and no denitrification to N_2_, but some accumulation of N_2_O, occurred from nitrite addition (Kaspar and Tiedje, 1981). Depending on the balance of enzyme activities, the reduction sequence from NO_3_^-^ to NH_4_^+^ can result in the accumulation of intermediates such as NO_2_^-^, NO, or N_2_O at any step (Wei, 2004). Normally, the reduction of nitrite to ammonia is much slower than the reduction of nitrate to nitrite, leading to the accumulation of nitrite. Thus, a dangerous concentration of nitrite may build up when a nitrate-rich diet is introduced to naïve livestock (Dawson et al., 1997).

Several major factors influence the toxicity of nitrite derived from nitrate (Leng, 2008; Lin et al., 2013b): (i) high nitrate concentrations in the diet, (ii) the rate of feed consumption, (iii) low rates of nitrite reduction to ammonia in the rumen, and (iv) slow rumen passage rate, resulting in longer nitrate, or nitrite retention in the rumen. *In vivo* studies have been careful to increase the dietary nitrate content gradually over a period of weeks to allow the rumen microbiota to adapt and for metabolism of nitrite to increase (Alaboudi and Jones, 1985; Nolan et al., 2010; van Zijderveld et al., 2010, 2011b). These enhanced nitrite reduction rates are of prime importance for increased safety with high-nitrate diets (Lin et al., 2013b). It could be speculated that nitrite, rather than nitrate, might be a better compound to use to induce this adaptation, because nitrate itself will enrich for enhanced nitrate reduction as well, but we were unable to find any published evidence of nitrite having been used in this way.

## Microbial Species Involved in Methanogenesis and Nitrate Reduction

The rumen is home to a vast array of ciliate protozoa, anaerobic fungi, anaerobic bacteria, and archaea, the complexity of which is beginning to be fully appreciated thanks to advances in molecular microbial ecology. The protozoa can comprise up to half the rumen microbial biomass, the fungi about 7%, the archaea 1–4% and the bacteria form the remainder and are normally the most abundant population. All contribute to methanogenesis in a direct or indirect way, but their role in and response to nitrate metabolism are much less clear.

### Archaea

**FIGURE 2:**
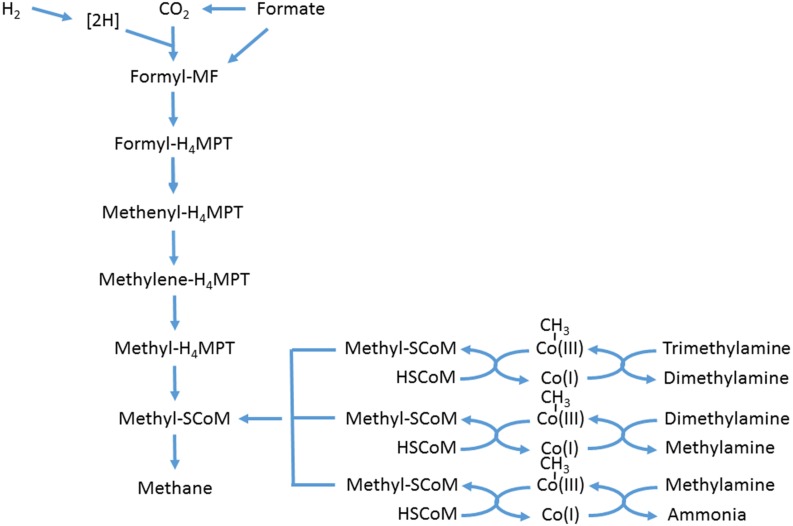
**Scheme of hydrogenotrophic and methylotrophic methanogenesis.** Adapted from Thauer et al. (2008), Rother and Krzycki (2010), and Shi et al. (2014). MF, methanofuran; MPT, tetrahydromethanopterin.

The ruminal methanogenic archaea comprise a narrow subset of the domain Archaea, in the sense that they are all methanogens, with the community dominated by *Methanobrevibacter* sp., which fall into two clades, one similar to *Mbb. ruminantium*, the other to *Mbb. gottschalkii* (Janssen and Kirs, 2008). Other significant genera include *Methanosphaera*, *Methanimicrococcus*, and *Methanobacterium* (Janssen and Kirs, 2008; Tymensen and McAllister, 2012; Kittelmann et al., 2013; Snelling et al., 2014). These archaea derive their energy by hydrogenotrophic methanogenesis, i.e., 4H_2_ + CO_2_ → CH_4_ + 2H_2_O. Formate can feed into the methanogenic pathway at the formyl level of the enzymatic sequence (**Figure 2**). It was only when the genome sequence of *Mbb. ruminantium* was analyzed that it was realized that an alcohol dehydrogenase was present, indicating that short-chain alcohols might be utilized by the hydrogenotrophic methanogens as well (Leahy et al., 2010). Another significant group of methanogenic archaea in the rumen has been known as Rumen Cluster C (RCC) (Janssen and Kirs, 2008), or *Methanoplasmata* [because they were found to be related to Thermoplasmatales (Poulsen et al., 2013)], or *Methanomassiliicoccus* (Dridi et al., 2012). The last group differ from the others in that they utilize methylamines, including tri-, di,- and monomethylamine, feeding into the methanogenic enzyme sequence at methyl-SCoM via methylcobamide:CoM methyltransferases (Rother and Krzycki, 2010; **Figure 2**). *Methanosarcina barkeri* has also been shown to use methylated amines (Patterson and Hespell, 1979). However, *Methanosarcina* have rarely been isolated from the rumen (Beijer, 1952; Rowe et al., 1979) and are almost never significant in molecular community analysis (Janssen and Kirs, 2008; Tymensen and McAllister, 2012; Kittelmann et al., 2013; Snelling et al., 2014). Although members of the domain Archaea do possess the nitrate reductase gene (Cabello et al., 2004), evidence for its existence in methanogenic archaea is lacking. The genome of *Mbb. ruminantium* contained no annotated nitrate reductase (Leahy et al., 2010).

How do the archaea respond to nitrate and products of its reduction such as nitrite? Community analysis of ruminal digesta from cattle or other ruminants receiving nitrate has so far been restricted to fairly broad characterization by ribosomal intergenic spacer analysis (Lin et al., 2013a) or qPCR (Asanuma et al., 2015) rather than more state-of-the-art 16S rRNA amplicon sequencing or metagenomics analysis. Both of the latter take advantage of developments in rapid, accurate DNA sequencing. rRNA amplicon sequencing enables the abundance of different members of the community to be determined in much greater detail and with far greater certainty than ribosomal intergenic spacer analysis or qPCR, and has been used to investigate, for example, rumen microbial community differences associated with animals that have low- or high-emitting methane phenotypes (Kittelmann et al., 2013). Metagenomic analysis enables the full gene profile that separates these phenotypes to be elucidated (Wallace et al., 2015). Clearly this is a major area for development in our quest to understand the effects of nitrate on methanogenesis. Available evidence from qPCR of archaeal 16S rRNA gene abundances suggests that archaeal abundance declined almost 10-fold in goats receiving nitrate (Asanuma et al., 2015), though methane emissions were not reported and effects on protozoa, fungi and to a lesser extent bacteria suggested a more general toxicity of the added nitrate in this study.

### Bacteria

The bacterial community of the rumen is very much more complex than that of the archaea. Although several thousand bacterial species may be present (Fouts et al., 2012), many will be transient, and a functional community of 200–400 species is likely to be present (Edwards et al., 2004; Jami and Mizrahi, 2012). Despite the large number of species, the predominant bacteria form only a narrow subset of the domain Bacteria, comprising mainly Bacteroidetes and Firmicutes, with smaller numbers of Proteobacteria and other phyla (Kim et al., 2011). Bacteria do not carry out methanogenesis, but are involved in the degradation of plant materials, which provides the substrates for methanogenesis by archaea, principally hydrogen. The main hydrogen producers are considered to be Firmicutes, particularly *Ruminococcus* sp. (Stewart et al., 1997). Although the vast majority of the bacterial community are strict anaerobes, many possess electron transport chains (Russell and Wallace, 1997) that can potentially be linked to nitrate reductase activity. Bacteria are generally considered to be primarily responsible for the reduction of nitrate and nitrite in the mixed ruminal community of adapted animals (Leng, 2008; Lin et al., 2011).

The bacterial species responsible for nitrate and nitrite reduction have been inferred by studying the bacterial communities in animals receiving nitrate, with some confirmation obtained by measuring nitrate/nitrite reductase activity *in vitro* in pure culture. Allison and Reddy (1983) made presumptive identification of *Selenomonas* sp. as predominant species from a continuous culture enrichment with nitrate, using an inoculum from nitrate-adapted sheep. Large Gram-positive rods formed another predominant group, but their identity is uncertain. Iwamoto et al. (2002) demonstrated by competitive PCR that *Veillonella parvula* (formerly *V. alcalescens*), and *Wolinella* (formerly *Vibrio*) *succinogenes* numbers were maintained in *in vitro* mixed culture if nitrate was supplied but otherwise they declined sharply. They also confirmed that *Selenomonas ruminantium* was active in nitrate and nitrite reduction, but only ssp. *lactilytica.* All three species reduced nitrate in pure culture (Iwamoto et al., 2002), with *W. succinogenes* possessing the highest activity (Iwamoto et al., 2002), and *V. parvula* having low nitrite reductase activity. The species mainly responsible for nitrate reduction changed with diet in the goat study of Asanuma et al. (2002). *S. ruminantium* was the most numerous of recognized species on both high-roughage and concentrate diets, and tended to be more abundant on the concentrate than on the roughage diet. Numbers of *W. succinogenes* and *V. parvula* were >10^4^-fold less than *S. ruminantium* (Asanuma et al., 2002). Asanuma et al. (2004) have purified the nitrate reductase from *S. ruminantium* and the gene was sequenced. The concentration of intracellular nitrate reductase-mRNA was higher when *S. ruminantium* was grown with nitrate than when grown without nitrate, suggesting induction by nitrate. Transcription of the nitrate reductase gene was also suggested to be enhanced in response to a deficiency of energy and electron supply. *Mannheimia succiniciproducens*, *V. parvula*, and *Campylobacter fetus* were obtained from nitrate enrichment culture and quantified by real-time PCR based on 16S rRNA sequence by Lin et al. (2013a). Nitrate supplementation increased the percentage of *C. fetus* and *M. succiniciproducens* in this study. Neither of these species is recognized as a predominant rumen species, however, and even after adaptation to dietary nitrate the relative population sizes were very low (<0.06% of 16S rRNA gene copy number). Asanuma et al. (2015) found that populations of methanogens, protozoa and fungi, as estimated by real-time PCR, were greatly decreased as a result of dietary nitrate inclusion, whereas *Streptococcus bovis* and *S. ruminantium* increased significantly. Yoshii et al. (2003) showed that the percentage of nitrate- and nitrite-reducing *S. ruminantium* in the total number of *S. ruminantium* was increased by feeding a high-nitrate diet added to a roughage diet for 12 weeks. Other studies in which increased numbers of nitrate-reducing bacteria or nitrate reductase activity were observed did not identify the specific bacterial species (Lin et al., 2011, 2013a). Thus, most evidence points to a very significant role for *S. ruminantium* in nitrate metabolism in the rumen.

Clearly, information on the ruminal bacteria that reduce nitrate and nitrite is very sparse and indeed is largely based on textbook properties (Stewart et al., 1997). Given the large number of species, including those that have not yet been cultivated (Kenters et al., 2011), many others may exist. This is a major gap in our knowledge that impacts upon our understanding of how nitrate might be used to mitigate methane emissions.

The other major issue is the species of ruminal bacteria that might be sensitive to the toxic effects of nitrate. Marais et al. (1988) showed that nitrite inhibited bacteria that produce ATP via electron transport systems, but had no effect on microbes that lack cytochromes and rely solely on glycolysis for ATP generation. Iwamoto et al. (2002) showed that growth of the three nitrate reducers was unaffected by nitrite, while 5 mM nitrite lowered, but did not eliminate, the growth of another 12 predominant species. As with the archaea, community analysis of ruminal digesta from cattle or other ruminants receiving nitrate has so far been restricted to fairly broad characterization by ribosomal intergenic spacer analysis (Lin et al., 2011) or qPCR (Asanuma et al., 2015) rather than more state-of-the-art 16S rRNA amplicon sequencing or metagenomics analysis. Cellulolysis by bacteria, in particular, is absolutely fundamental to optimally productive rumen fermentation. Therefore, it is important that we understand how the cellulolytic population responds to dietary nitrate, particularly as some of the key species seem to be sensitive to nitrate and its more reduced intermediates. Once again, this is a major gap in our understanding of nitrate as a feed additive to lower methane emissions.

### Ciliate Protozoa

Rumen ciliates are eukaryotic microorganisms that are visible to the naked eye, because they may reach 200 μm in length (Williams and Coleman, 1997). Over 250 ciliate species have been described from various ruminants (Williams and Coleman, 1992). They can be divided into two orders in the class Trichostomatida, Vestibuliferida, and Entodiniomorphida (Small and Lynn, 1981). Similar species inhabit the digestive tract of various vertebrates, and almost all the members of Entodiniomorphida inhabit the rumen or large intestine of large herbivorous mammals. Metabolically, the protozoa are rather similar to bacteria in the substrates used and products formed (Williams and Coleman, 1997). However, they differ in that they possess a cytoplasmic organelle, the hydrogenosome, which has evolved from mitochondria (Embley et al., 2003). As its name implies, the hydrogenosome forms hydrogen, and it contains electron transport carriers that might conceivably relay electrons during nitrate reduction. The ciliate protozoa, because they produce abundant amounts of hydrogen, form a central component of substrate supply for methanogenesis. This is reflected in the intimate association between ciliates and archaea. Archaea can be seen to colonize the outer surface of protozoa (Vogels et al., 1980) and remarkably also occur as endosymbionts in the cytoplasm, presumably because of high local concentrations of hydrogen in proximity to hydrogenosomes (Finlay et al., 1994).

How important are protozoa in nitrate metabolism in the mixed ruminal community? Rumen protozoa were reported to accelerate nitrate reduction when co-cultured with bacteria (Yoshida et al., 1982). The protozoal fraction had greater ability for nitrate and nitrite reduction than the bacterial fraction, and inhibition of methane production by nitrate was greatest in the protozoal fraction. Similar results were obtained by Lin et al. (2011), but furthermore it was shown that nitrate metabolism by the protozoal fraction did not result in the accumulation of nitrite, whereas nitrite accumulated in incubations with the bacterial fraction. Both these studies confirm that protozoa play an integral part in nitrate metabolism, and indeed may be vital for the safe use of nitrate because of their activity in reducing nitrite. Furthermore, there may be a symbiotic relationship between protozoa and associated bacteria, whereby both reduce nitrate and the protozoa mainly reduce nitrite (Lin et al., 2011).

How does the ciliate protozoal community respond to nitrate and its reduction products? Several papers suggest a negative effect. Sar et al. (2005a) noted a fall in protozoal numbers in sheep receiving nitrate. Asanuma et al. (2015) found that protozoal abundance fell by >86% in goats receiving 6 g of potassium nitrate per day, and cited another of their studies in which a similar decrease was observed. In contrast, van Zijderveld et al. (2010) reported that protozoal numbers were unaffected. Given that protozoa may have a crucial role in the safe use of nitrate as a feed additive, we need to know much more about protozoal metabolism of nitrate and nitrite and their response to dietary nitrate.

### Anaerobic Fungi

The other main category of eukaryotic microorganism in the rumen is the anaerobic fungi. Perhaps 20 different species are present, all belonging to the phylum Neocallimastigomycota (Orpin and Joblin, 1997; Gruninger et al., 2014). Indeed variations in ITS regions of ribosomal RNA genes rather than the hypervariable regions within the genes themselves are used to distinguish the different taxa (Fliegerova et al., 2004). Anaerobic fungi comprise perhaps 0–8% of rumen microbial biomass (Orpin and Joblin, 1997). Their main function is plant fiber breakdown, indeed they are the only rumen cellulolytic species that physically as well as enzymically degrade plant fiber (Ho et al., 1988). Thus they are particularly valuable to animals consuming poorer quality forages (Gordon and Phillips, 1993). The fungi, as do the protozoa, possess hydrogenosomes, and they are significant producers of hydrogen (Marvin-Sikkema et al., 1993, 1994).

Lin et al. (2011) found that the nitrate-reducing activity of a fungal fraction from ruminal digesta was low, so their contribution to nitrate metabolism is likely to be minor. The main concern if fungi are generally suppressed by nitrate would be reductions in fiber digestion, particularly of the more recalcitrant plant cell walls.

## Mechanisms of Inhibition of Methanogenesis by Nitrate

**FIGURE 3:**
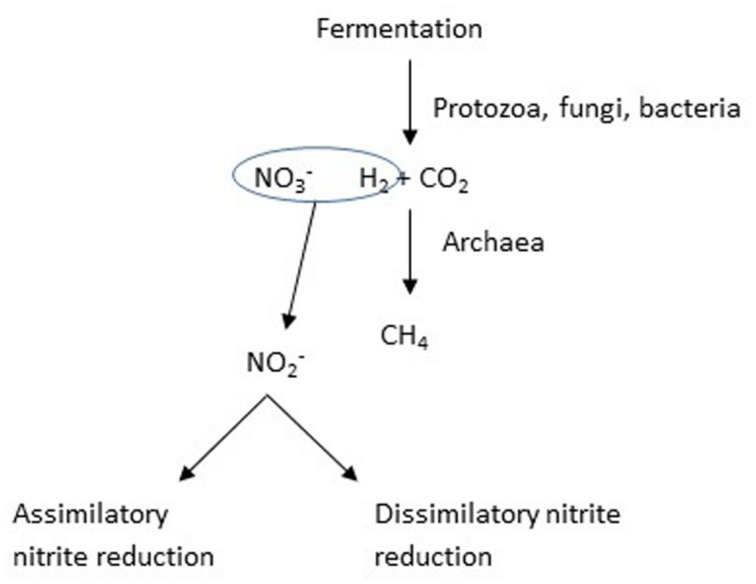
**The addition of nitrate is intended to provide an alternative hydrogen sink, in other words a competition for available hydrogen**.

When Jones (1972) observed decreased methane production in response to nitrate, the effect was interpreted as a possible consequence of nitrate raising the redox potential, E_h_, of the medium; however, most subsequent studies have considered nitrate to be an alternative hydrogen sink to methane production (**Figure 3**). Methane is produced in the rumen predominantly by the hydrogenotrophic route, whereby hydrogen and carbon dioxide are the principal substrates (Hungate, 1967). Several compounds or their metabolites that could act as alternative hydrogen sinks to methane have been identified, including sulfate and propionate precursors like fumarate and acrylate (Newbold et al., 2005), but with a few exceptions, these have been relatively inefficient, in the sense that the efficiency of trapping of hydrogen has been low. In contrast, Iwamoto et al. (1999) found that the efficiency of nitrate in inhibiting methane production was high, but this may have occurred because of the toxicity of the nitrite that accumulated. The thermodynamics of trapping hydrogen by nitrate reduction are favorable (Klüber and Conrad, 1998).

The alternative hydrogen sink hypothesis is used most often to explain how nitrate lowers methane production in anaerobic ecosystems. There may be other mechanisms involved as well, however. N-oxide intermediates, such as nitrite and nitrous oxide, may suppress methanogenesis directly (Ungerfeld, 2015). Evidence for this mechanism was provided by Klüber and Conrad (1998) using anoxic slurries of Italian rice soil, in which “Especially after addition of nitrite and NO, toxic effects may have been more important than competition.” The toxic effects were speculated to be disturbance of redox balance in microbial cells, but direct inhibition was not ruled out. As methanogens do not contain menaquinone or appreciable amounts of b- or c- cytochromes, and obtain energy exclusively by electron transport-linked phosphorylation (Thauer et al., 1977), inhibition of methanogens by nitrite at the electron-carrier system is suggested.

Other possible impacts may arise from the toxicity of nitrate or its products to ruminal microorganisms, altering their metabolism, particularly hydrogen production. If the sensitive species are hydrogen producers, methane production may be decreased by the lower supply of hydrogen. In support of such a mechanism, Marais et al. (1988) noted that *Ruminococcus* sp., which are important hydrogen producers in the ruminal ecosystem (Stewart et al., 1997), were extremely sensitive to low concentrations of nitrite – growth was inhibited at 2 mg nitrite-N/l. However, other, non-hydrogen producing bacteria were equally sensitive to nitrite. In contrast, Iwamoto et al. (2002) found that, although growth of 12 species of ruminal bacteria was suppressed by nitrite, in no instance was growth prevented. Clarification of the relative sensitivity of different microbial species to the products of nitrate reduction and their ability to adapt their metabolism would greatly enhance our understanding of the mechanism of action of nitrate in inhibiting ruminal methanogenesis.

Although hydrogen and carbon dioxide are the predominant substrates for methanogenesis, others are present too. The relatively recently described group of *Thermoplasmata* (Poulsen et al., 2013; now named as a family Methanoplasmatales, Shi et al., 2015) metabolize methylamines. Trimethylamine is formed from trimethylglycine and choline. The former is particularly abundant in beet pulp, which was a component of the diet used by Poulsen et al. (2013). It would be instructive to determine if nitrate or its reduction products influence methanogenesis from methylamines. If the main mode of action is disruption of the redox status of key cellular electron carriers, presumably methanogenesis from methylamines would be affected as for the hydrogenotrophic methanogens.

Thus, although the hydrogen sink is usually considered to be the mechanism whereby nitrate inhibits ruminal methanogenesis, future studies to develop nitrate as a feed additive must take account of the relative importance of each of the other potential mechanisms, in order to be sure that the perceived mechanism, a possible target for manipulation, is correct. The reason for finding out what is the real mechanism is quite simple. Four moles of hydrogen are consumed to produce one mole of methane. At best, assuming completion of the assimilatory pathway, four moles of hydrogen will be used to convert nitrate to ammonia. Thus to theoretically capture the quantities of hydrogen that are normally converted to methane, a dietary inclusion of 80–100 g nitrate/kg dietary dry matter would be required for a productive dairy cow or growing steer. This inclusion of nitrate is not practicable because of risks of methemoglobinemia alone, and practical inclusions are around 20 g nitrate/kg dry matter. These lower additions of nitrate in dairy cows gave a 16% reduction in methane output but no production benefit (van Zijderveld et al., 2011b). This reduction might be consistent with either mechanism. More studies need to be done to determine if lower amounts of nitrate may be adequate to have a significant inhibitory effect on methanogenesis.

## Impact of Nitrate on Other Metabolic Activities of the Ruminal Microbiota

The thermodynamically favorable reduction of nitrate preferentially directs hydrogen away from methanogenesis, but could also draw hydrogen away from other processes such as propionogenesis (van Zijderveld et al., 2010) and fatty acid biohydrogenation (Lourenço et al., 2010), and influence other areas of fermentation unfavorably.

In terms of the formation of the main fermentation products, the VFAs are key nutrients for the host animal. Farra and Satter (1971) observed a shift in the VFA profile from propionate to acetate when diets high in nitrate were fed to dairy cows. The butyrate concentration was also significantly lowered. The same phenomenon has been observed in many other studies (Lee and Beauchemin, 2014), although butyrate concentration increased when nitrate was added to the diet in the beef study of Troy et al. (2015). The explanation for this change in propionate is that nitrate is an alternative electron acceptor to endogenous fumarate in many propionate-producing bacteria (see below). Thus, nitrite is formed rather than succinate, which would then be decarboxylated to propionate, and the balance of VFA moves away from propionate. Following absorption, propionate is the only VFA that is glucogenic, so a lower molar propionate production rate would generally be considered to be detrimental to nutrition (Leng et al., 1967).

The rumen evolved as an organ whereby the passage through the gut of plant fiber is retarded, enabling fibrolytic microorganisms more time to degrade cellulose, hemicellulose, and pectin polymers. Fiber breakdown is key to the efficient utilization of forage feedstuffs. Marais et al. (1988) found that cellulolytic ruminococci were sensitive to quite low concentrations of nitrite, and Iwamoto et al. (1999) found that nitrite suppressed fermentation in general. However, a major survey concluded that, if adaptation was conducted carefully when nitrate was introduced, no detrimental effects on feed intake or weight gain would occur (Lee and Beauchemin, 2014).

## Improving the Safety and Efficacy of Nitrate Supplementation

### Safety

In monitoring animal health by measuring blood methemoglobin, mean values are normally reported. However, the key indicator is not the mean but the extreme response. The death of one or two animals is a much greater loss than a depression in average performance. Extreme responders do exist even when animals are gradually adapted to feeding nitrate. Cockrum et al. (2010) classified sheep into groups with low and high tolerance to nitrate; Newbold et al. (2014) removed cattle from an experiment because of high methemoglobin concentration; Lee et al. (2015b) identified specific animals which did or did not increase blood methemoglobin in response to dietary nitrate. Similarly, when Duthie et al. (2015) adapted cattle gradually to nitrate and repeat tested over 70 days, blood methemoglobin was consistently elevated in specific cattle. Duthie et al. (2015) also noted (in agreement with Lee and Beauchemin, 2014) that there was no relation between blood methemoglobin and animal performance and so in adapted animals elevated methemoglobin may not necessarily indicate adverse health consequences. Individual animals also vary in the extent of methane reduction when fed nitrate; Troy et al. (2015) recorded a 17% mean reduction in methane when nitrate was fed, but individual animal response ranged from 0 to 28% reduction. Understanding individual animal responses to nitrate, including both animal and microbial components, is necessary to improve safety.

**FIGURE 4:**
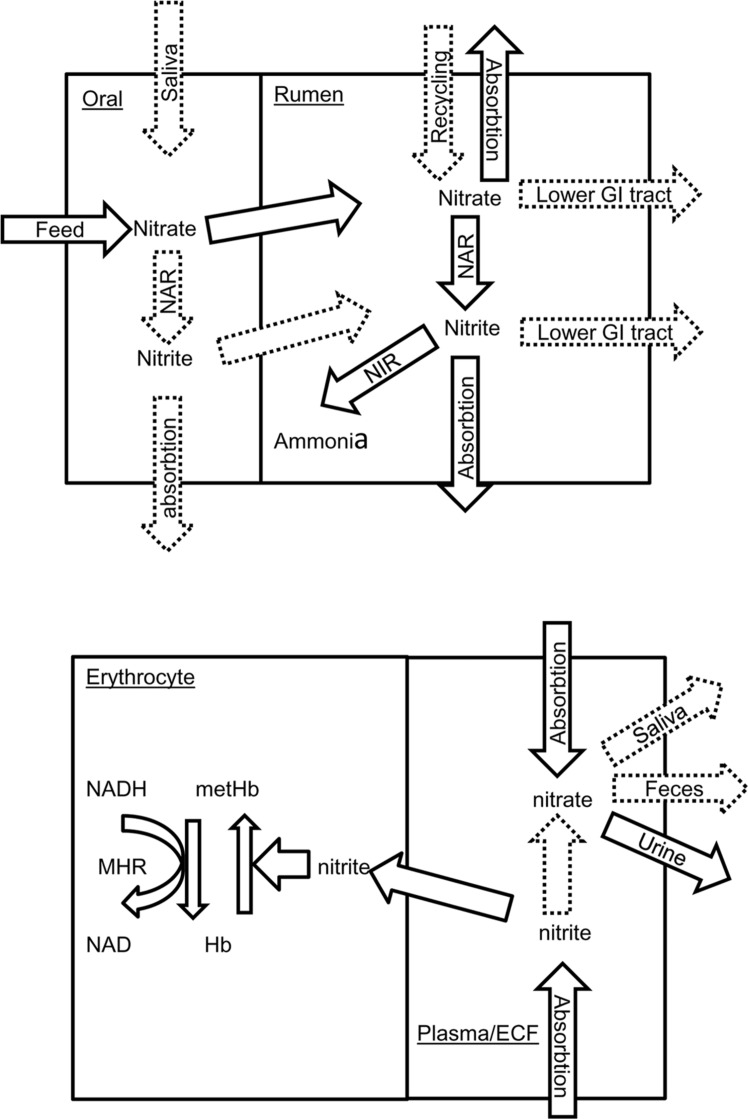
**Simplified flow diagram showing nitrate and nitrite utilization in the ruminant oral cavity and rumen (top) and erythrocyte and blood plasma/extracellular fluid (ECF) (bottom).** Documented processes are show as solid arrows whilst those inferred are shown as broken arrows. NAR, nitrate reductase; NIR, nitrite reductase; GI, gastrointestinal tract; Hb, hemoglobin; met Hb, methemoglobin; MHR, NADH-dependent methemoglobin reductase.

It is increasingly clear there is a complex interaction between the rumen microbiome, diet, and host animal and that host genotype influences the rumen microbiome (King et al., 2011; Hernandez-Sanabria et al., 2013), at least partly explaining variation in individual animal response. For methemoglobin, specific animal factors that influence concentrations include rates of feed consumption, nitrite absorption from the rumen, re-oxidation of nitrite to nitrate within animal tissues, oxidation of methemoglobin to hemoglobin and recycling of nitrate to the rumen. As indicated in **Figure 4**, information is sparse on many of these factors and to improve safety when feeding nitrate, the critical factors require identification. For example, although nitrate can be recycled to the rumen (Leng, 2008), it is not known if nitrate is concentrated into saliva from plasma as in humans (Cockburn et al., 2013). Careful adaptation of ruminants to nitrate-containing diets may not only allow the rumen microbiome to adapt but also the host animal. Godwin et al. (2015) reported increased erythrocyte methemoglobin reductase activity when cattle were fed nitrate. As inorganic phosphate also increases erythrocyte methemoglobin reductase activity, ensuring adequate dietary phosphorus in nitrate-fed animals may improve clearance of blood methemoglobin. The animal factor most amenable to manipulation is rate of feed intake. Conditions which encourage rapid feed consumption such as restricted vs. ad libitum feeding are associated with higher methemoglobin and nitrate poisoning (de Raphélis-Soissan et al., 2014; Lee et al., 2015b); cattle fed nitrate change their feeding pattern (decreased feed consumption, Lee et al., 2015b); increased number of small meals, Velazco et al., 2014) to reduce the risk of methemoglobin formation. Practical feeding strategies should avoid situations that encourage rapid feed consumption.

The type of diet offered may influence rates of rumen nitrate and nitrite reduction. Tillman et al. (1965) reported that lower rumen pH (<6.0) favored nitrite reduction and prevented nitrite accumulation; however, low rumen pH was achieved by feeding sheep molybdenum-deficient diets. As nitrate reductase is a molybdenum pterin cofactor-containing enzyme (Magalon et al., 2011), the effects on nitrite accumulation ascribed to pH by Tillman et al.(2015) could be confounded by molybdenum deficiency and therefore are not reliable. In contrast, Iwamoto et al. (2001a) found greater nitrate and nitrite reduction at pH 7.0 than 6.0. In agreement, blood methemoglobin (Troy et al., 2015) was greater on high-concentrate diets, which are associated with lower rumen pH. Because of the greater bulk of high forage diets, slower feeding rates on these diets compared to high concentrate diets, together with higher rumen pH, may be important in avoiding methemoglobin accumulation.

Gradual introduction of nitrate to the diet is intended to decrease nitrite accumulation through enhancement of the kinetics of nitrite reduction to ammonia and hence toxic methemoglobin concentrations. Typically, dietary nitrate is increased step-wise over time. However, the total length of time to adapt animals to nitrate varies widely as does time on each step: 7–21 day total adaptation time and 2–7 day on each step (van Zijderveld et al., 2011b; Li et al., 2012; El-Zaiat et al., 2014; Lee et al., 2015a). The optimal strategy based on comparison of responses does not seem to have been investigated. There is a need to establish minimum conditions for successful adaptation to nitrate-containing diets. During adaptation, the rates of nitrate and nitrite reduction (Alaboudi and Jones, 1985; Lin et al., 2013a), activity of nitrate reductase (Asanuma et al., 2015) and numbers of nitrate-reducing bacteria increase (Lin et al., 2013b). However, the rate of nitrite reduction remains less than that of nitrate reduction, thus still favoring nitrite accumulation. The reason for successful adaptation may be that increased rates of nitrate and nitrite reduction increase net conversion of nitrate to less toxic compounds such as ammonia. To minimize risk to the animal, the rate of nitrite reduction should be greater than that of nitrate.

Increased nitrite reduction may be achieved nutritionally, by manipulating the rumen microbiome or by introducing specific novel microorganisms. Electron donors, such as formate and lactate, when included together with nitrate *in vitro*, increased nitrite reduction and reduced methane production to a greater extent than for nitrate alone (Iwamoto et al., 2001a; Yoshii et al., 2005). However, the fermentation of diets including cellulose, hemicellulose, and starch which are normally fed to productive livestock will produce lactate and formate as intermediates or end-products of fermentation. *In vivo*, therefore, any benefits in increased nitrate reduction resulting from increased lactate and formate supply by alternative nutritional strategies are likely to be limited.

Adding sulfate to nitrate-containing diets might lower nitrite concentrations, as sulfate-reducing bacteria, specifically *Desulfovibrio* species, are able to reduce nitrite *in vitro* but not nitrate (Mitchell et al., 1986). Methane production was also decreased both *in vitro* (Patra and Yu, 2014; Wu et al., 2015) and *in vivo* (van Zijderveld et al., 2010; Li et al., 2013) in response to sulfate. Methemoglobin was not detected when sulfate was added to a nitrate-containing diet (van Zijderveld et al., 2010) compared with low levels of methemoglobin detected when nitrate alone was added; the small scale of the experiment, however, precludes concluding that dietary sulfate reduced the occurrence of methemoglobinemia. In addition, high dietary sulfur intakes can be toxic inducing polioencephalomalacia and therefore caution must be exercised in the use of dietary sulfate (Drewnoski et al., 2014).

Introducing microorganisms containing nitrite reductase into the rumen may increase nitrite reduction. Administration of *Propionibacterium acidipropionici* was without effect (de Raphélis-Soissan et al., 2014). However wild type *Escherichia coli* (but not a genetically modified strain with enhanced nitrite reductase activity) lowered rumen nitrite and blood methemoglobin concentrations (Sar et al., 2005a); this promising response was to a single dose of *E. coli* and long term adaptive responses require evaluation. Sakthivel et al. (2012) found that an unidentified nitrate-reducing rumen bacterium enhanced nitrate and nitrite removal from ruminal digesta *in vitro* and decreased methane formation. In general, a lack of information about the members of the rumen microbiome responsible for nitrate and nitrite reduction and how the microbiome changes when nitrate is included in the diet restricts attempts to manipulate the microbiome to enhance nitrate and nitrite reduction. For example, protozoa may be responsible for a substantial proportion of nitrate reduction in the rumen (Lin et al., 2011). However, this study used ruminal fluid from animals not adapted to nitrate. Recently, Asanuma et al. (2015) quantified microbial abundance by 16s rRNA gene sequencing and found that protozoal populations declined sevenfold in goats adapted to dietary nitrate, suggesting protozoa may be of lesser importance. It will be vital to analyze the ruminal microbiome in detail in order to understand the microbiological basis for different responses.

### Efficacy

Although the energetics of reducing nitrate to ammonia via the dissimilatory pathway are more favorable than converting hydrogen and carbon dioxide to methane, *in vivo* decreases in methane emissions are less than would be expected when stoichiometrically 1 mol (62 g) of nitrate, fully reduced to ammonia in the rumen, should lower methane formation by 1 mol (16 g). Leng (2014) reviewed published studies and demonstrated a negative relationship between dietary nitrate and methane emissions such that methane emissions declined from 100 to 60% of the theoretical maximum as nitrate inclusion (g/kg diet dry matter) increased. Several explanations are possible for lower than expected decreases in methane emissions. Firstly, nitrate and/or nitrite may be absorbed from the rumen and excreted rather than reduced to ammonia if the rate of feed nitrate ingestion is greater than the capacity for reduction. Lee et al. (2015a) found that 1.5–3% of nitrate consumed was recovered in feces and urine when the mean decrease in methane emissions was 51% of the maximum possible. After correction for nitrate in feces and urine, the mean decrease in methane emissions was 56% and thus nitrate excretion only explained a small proportion of the lower than expected decrease in methane emissions. Second, nitrite may be metabolized to end-products other than ammonia such as nitrogen and nitrous oxide gasses. Since the environmental objective of feeding nitrate is to lower GHG emissions, then production of nitrous oxide, which has a global warming potential more than 10 times greater than methane, is not desirable. de Raphélis-Soissan et al. (2014) measured nitrous oxide production when nitrate was fed to sheep and nitrous oxide emissions were increased which, when accounted for, reduced the GHG benefits of nitrate feeding from 80 to 68 g carbon dioxide equivalent/kg dry matter intake. Clearly nitrous oxide emissions must be accounted for when assessing the benefits of feeding nitrate. Thirdly, feeding nitrate might, by lowering feedback inhibition, increase total hydrogen production and thus the effect of nitrate on methane production would be less than predicted.

Research should be directed to maximizing decreases in methane emissions for a given intake of nitrate. It is also important to ensure maximum conversion of nitrate to ammonia, the primary substrate for microbial protein synthesis so that nitrate can replace dietary rumen degradable protein sources analogous to the use of urea. Nitrate should not be added to diets already adequate in rumen-degradable nitrogen supply as excretion of excess nitrogen can lead to increased nitrous oxide production from soil after manure application. In many experiments, nitrate intakes have been balanced by inclusion of urea in control diets (Lee and Beauchemin, 2014); animal performance on the nitrate and urea-containing control diets have been similar. However, there is little evidence concerning animal performance on nitrate-containing diets compared with control diets where nitrate replaces protein rather than urea. If less than 100% of nitrate consumed is converted to ammonia, ammonia supply to the rumen will be less than for diets containing nitrogenous substrates completely available to the rumen microbial community. There is a need for experimental designs which include a negative control treatment for dietary rumen degradable protein so that nitrate and urea supplementation can be compared.

If conditions for feeding nitrate which achieve both maximum conversion of nitrate to ammonia and lowering of methane production limit the amount of nitrate that can be fed, then an alternative approach is to use nitrate in combination with other strategies known to lower methane emissions. Combining strategies for lowering methane emissions with different mechanisms has scarcely been investigated. Iwamoto et al. (1999) found that using both fumarate and nitrate was beneficial. Addition of nitrate and fumarate did not affect intake, nutrient utilization, microbial protein supply, and blood profile (Pal et al., 2015). Patra and Yu (2013) in an *in vitro* study, found that combining inhibitors of methane production with complementary mechanisms at low doses could be more effective and practical in mitigating methane emissions from ruminants without impairing feed digestion. Combination of saponins and nitrate may be such a practical strategy. Patra and Yu (2014) showed *in vitro* that combinations of nitrate with saponins and sulfate additively suppressed methane production, with the maximum reduction in emissions (nearly 46%) observed for the combination of three inhibitors. When sulfate and nitrate were fed to sheep (van Zijderveld et al., 2010), the effects of sulfate and nitrate on methane production were additive, indicating potential for this combined approach. Of course, there is a need for long-term performance experiments with large numbers of animals to better assess persistency of single- or combination-strategy approaches to methane mitigation on feed intake, performance, meat, and milk characteristics.

## Likely Consequences of Inhibiting Methanogenesis on Productivity

The possible consequences of a successful outcome to current ruminant methane research have prompted much discussion and some experimental and data analysis. On the one hand, it would seem to be intuitive that decreasing the loss of an energy-rich product, methane, would enhance energy retention within the animal’s body and thereby enhance nutritional efficiency. Thermodynamic considerations would support such a view (Ungerfeld, 2015). On the other, it has been widely believed for many years that the elimination of methanogenesis would lead to an accumulation of the substrate gas, hydrogen, which is a product of fermentation by acetate and butyrate producing microorganisms, and that this accumulation would suppress fermentation rates in the rumen (Wolin et al., 1997), particularly in microenvironments (Leng, 2014). This belief was founded mainly upon pure-culture studies in which hydrogen accumulation by a single H_2_-producing bacterial species resulted in thermodynamic inhibition of fermentation and growth (Iannotti et al., 1973; Latham and Wolin, 1977; Wolin et al., 1997). Co-culture with a methanogen relieved this inhibition. As the main cellulolytic species are hydrogen producers, it was feared that preventing methane emissions would lead to H_2_ accumulation which would in turn slow fiber breakdown. The effects of hydrogen concentration are in fact much more complex (Janssen, 2010). Studies in gnotobiotic lambs lacking methanogens (Fonty et al., 2007) and inhibiting methane emissions in goats and cattle using experimental halogenated compounds (Mitsumori et al., 2012) suggested that growth was normal and other effects such as on feed intake were minor. Further, as yet unpublished experiments with cattle confirm such an outcome, with the animals emitting hydrogen gas instead of methane (S. C. Denman, personal communication). An overall benefit, due to mitigation of methanogenesis by dietary nitrate supplementation, on ruminant animal energetics has not been detected (van Zijderveld et al., 2011a; Knapp et al., 2014; Hristov et al., 2015). Additional research in this area will affirm or refute this preliminary conclusion.

In summary, it is clear that we would benefit from further research in several areas to ensure consistent and safe use of nitrate as a means of mitigating methane emission and this review suggests that the most important questions are:

• How does the microbial community, as measured by contemporary methodology, respond, and how can that response be improved to enable safe adaptation to nitrate consumption? Differences in microbial community between animals that successfully adapt to nitrate and those that do not adapt should reveal the answer. The sensitivity of cellulolytic bacteria is of special interest.
• What is the role of ciliate protozoa in nitrate metabolism: what is the significance of their nitrate reduction *in vivo*? If it is substantial, does nitrate reduction lower methane emissions from symbiotic archaea?
• Which bacterial species, other than those already recognized, use nitrate and which are sensitive to the toxic properties of nitrate and especially nitrite?
• Is nitrate really a good source of non-protein N for microbial growth?
• What are the mechanisms by which methane production is suppressed? Is the hydrogen sink hypothesis the main mechanism?
• What are the relative rates of flux through the nitrate reduction pathway in adapted and non-adapted animals, in order to avoid the accumulation of unwanted intermediates?
• What is the basis of the inhibitory effect of nitrate on methanogenesis and on the changes in ruminal fermentation in animals which are gradually adapted to the nitrate diet?
• Long-term performance experiments with large numbers of animals are encouraged to better define effects of nitrate in combination with other strategies for mitigating methane production on feed intake animal performance, and meat and milk characteristics.

## Author Contributions

All authors wrote sections of the paper. RW prepared the final manuscript. All authors read and approved the final manuscript.

## Conflict of Interest Statement

The authors declare that the research was conducted in the absence of any commercial or financial relationships that could be construed as a potential conflict of interest.
